# Discrepancies between community interpretations and emergency radiology re-interpretation of imaging exams on trauma patients transferred to a level 1 trauma center

**DOI:** 10.1007/s10140-025-02421-7

**Published:** 2025-11-28

**Authors:** Allison Crone, Josh VanZant, Alexander Boutselis, Scott D. Steenburg

**Affiliations:** 1 Department of Radiology and Imaging Sciences, Indiana University School of Medicine, 550 N. University Blvd. University Hospital Building Room 0663, Indianapolis, IN 46202 USA; 2 Indiana University School of Medicine, Indianapolis, IN USA; 3 Department of Radiology, Mayo Clinic, Phoenix, AZ USA

**Keywords:** Trauma, Discrepancy, Medical error

## Abstract

**Purpose:**

To evaluate the rate of discrepancies between community radiologists’ interpretations of trauma studies and those of the emergency radiologists at an academic level 1 trauma center and to assess the impact on patient management provided by emergency radiologist overreads.

**Methods:**

A search of our institutional Radiology Information System (RIS) was performed to find outside overread interpretations that were performed by emergency radiologists at our institution in a 1-year period (January 1, 2023-December 31, 2023). For these patients, the outside report was obtained and compared to the emergency radiologist overread report. Discrepancies between reports were divided into major and minor discrepancies. A major discrepancy was defined as a change in interpretation that resulted in altered patient management (including interventional radiology procedure or surgery). A minor discrepancy was defined as a change in interpretation that did not alter patient management.

**Results:**

The study cohort consisted of 301 imaging exams performed on 135 patients. The mean age was 52.3 years (range 19–97 years), included 68 males (63.7%), and a mean Injury Severity Score (ISS) of 13 (range 1–51). Patients were transferred from 29 different facilities from around our state. The most common mechanism of injury was fall (*n* = 128, 42.5%) followed by motor vehicle collisions (MVC, *n* = 103, 34.2%) and assaults (*n* = 25, 8.3%). Major discrepancies were found in 26 exams (8.6%) affecting 25 patients (18.5%), of which 7 required surgery or an interventional radiology procedure. Minor discrepancies were found in 49 exams (16.3%) affecting 42 patients (31.1%). In total, some discrepancy was found in 75 (24.9%) of exams affecting 62 patients (49.6%). There is a statistically significant association with a discrepancy following MVC compared to the other mechanisms of injury (*p* = 0.0228).

**Conclusions:**

We found that nearly a quarter (24.9%) of overread imaging exams affecting nearly half (49.6%) of transferred trauma patients had a discrepancy in the original report, resulting in a change in management in 18.5% of patients, with over a quarter (26.9%) of major discrepancies requiring surgery or interventional radiology procedures. Motor vehicle collisions were associated with an increased likelihood of a discrepancy. Our findings continue to support the value of reinterpretation of outside imaging exams by dedicated emergency radiologists in the setting of trauma.

## Introduction

Reinterpretation of imaging exams (also known as “overreads” or “double reads”) is a common practice when transferring patients to tertiary care centers for specialty care, and is performed in many clinical settings, including oncologic conditions, surgical complications, or trauma care [1–4]. Previous studies have noted the discordance of radiologic interpretations of community radiologists and dedicated emergency radiologists, with reported discrepancy rates approaching 20% when reinterpreted by a tertiary care referral center emergency radiologist [3, 5–10]. Our institution has seen an increasing number of overread requests for imaging performed on trauma patients who are transferred to our adult level I trauma center. This study was conducted to assess the value added by performing these reinterpretations at our institution, as compared to the initial finalized report from the originating institution with a specific interest in alterations in management. We also analyzed factors that could potentially be associated with an increased likelihood of discrepancy.

## Methods

All patients transferred to our institutional adult level I trauma center during a single year period (1/1/2023 to 12/31/2023) were considered for study inclusion. Only exams that were performed on trauma patients and had a finalized overread report by a member of our emergency radiology division were included for analysis. Patient demographics, mechanism of injury (MOI), Injury Severity Score (ISS), and originating institutions were recorded. Studies without access to the initial report from the originating institution were excluded from analysis.

The included studies were assessed for the presence and significance of any discrepancies between the original report and the overread report. Reports were categorized into 3 groups: no discrepancy, defined as no clinically significant differences between the two reports; minor discrepancies, defined as the presence of a difference in interpretation that that did not alter patient management; and major discrepancies, defined as the presence of a difference in interpretation that altered patient management, including (but not limited to) additional confirmatory or clarifying diagnostic tests, change in medical management, interventional radiologic procedure, or surgery. The primary author (AC, who was a senior diagnostic radiology resident at the time of analysis) made these determinations by comparing the original outside interpretation with the overread final report in conjunction with a review of the medical record to determine patient management. The senior author (SS, a trauma/critical care radiology fellowship trained radiologist with 15 years of experience) adjudicated any equivocal cases.

## Results

During the one-year study period, a total of 266 patients were transferred to our level 1 trauma center, resulting in 506 imaging studies that were overread by an emergency radiologist. Of these patients, 166 patients with 383 imaging studies were for the indication of trauma. One hundred thirty-five patients with 301 imaging studies that had an outside report available for review constituted the study cohort.

Of the 135-patient cohort, 68 were male (63.7%), the mean age was 52.3 years (range 19–97 years), and the mean Injury Severity Score (ISS) was 13 (range 1–51). Patients were transferred from 29 different facilities from around our state.

The most common mechanism of injury was fall (*n* = 128, 42.5%) followed by motor vehicle collisions (MVC, *n* = 103, 34.2%) and assault (*n* = 25, 8.3%), detailed in Table 1.

**Table 1:** Mechanisms of injury and number of exams

Mechanism of injury	Number of exams (%)
Fall	128 (42.5%)
MVC	103 (34.2%)
Assault	25 (8.3%)
MCC	15 (5.0%)
Other blunt mechanism NOS	12 (4.0%)
ATV	8 (2.7%)
GSW	4 (1.3%)
Bicycle accident	3 (1.0%)
Crush injury	2 (0.7%)
Stab wound	1 (0.3%)

*MVC* motor vehicle collision, *MCC* motorcycle collision, *NOS* not otherwise specified, *ATV* all terrain vehicle, *GSW* gunshot wound

**Fig. 1:**
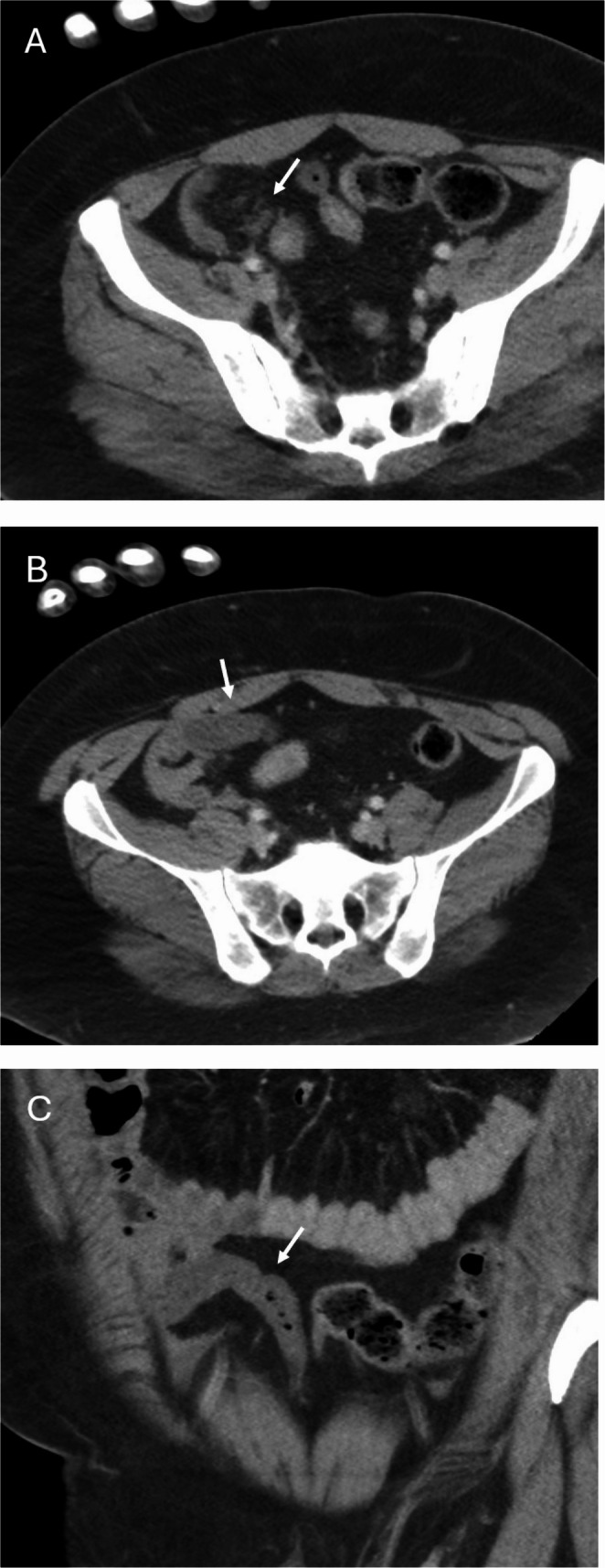
27-year-old male with abdominal pain status-post motor vehicle collision. Axial (**A**, **B**) and coronal (**C**) postcontrast CT images through the pelvis demonstrate a subtle focus of mesenteric fat stranding (A, arrow) in the right lower quadrant indicative of a mesenteric injury, which was not described on the original outside report. There is an adjacent loop of poorly enhancing small bowel (B & C, arrows) which also was not described on the original outside report. At surgery, there was a small bowel mesenteric bucket handle injury associated with devitalized small bowel. A total of 30 cm of small bowel was resected.

**Fig. 2:**
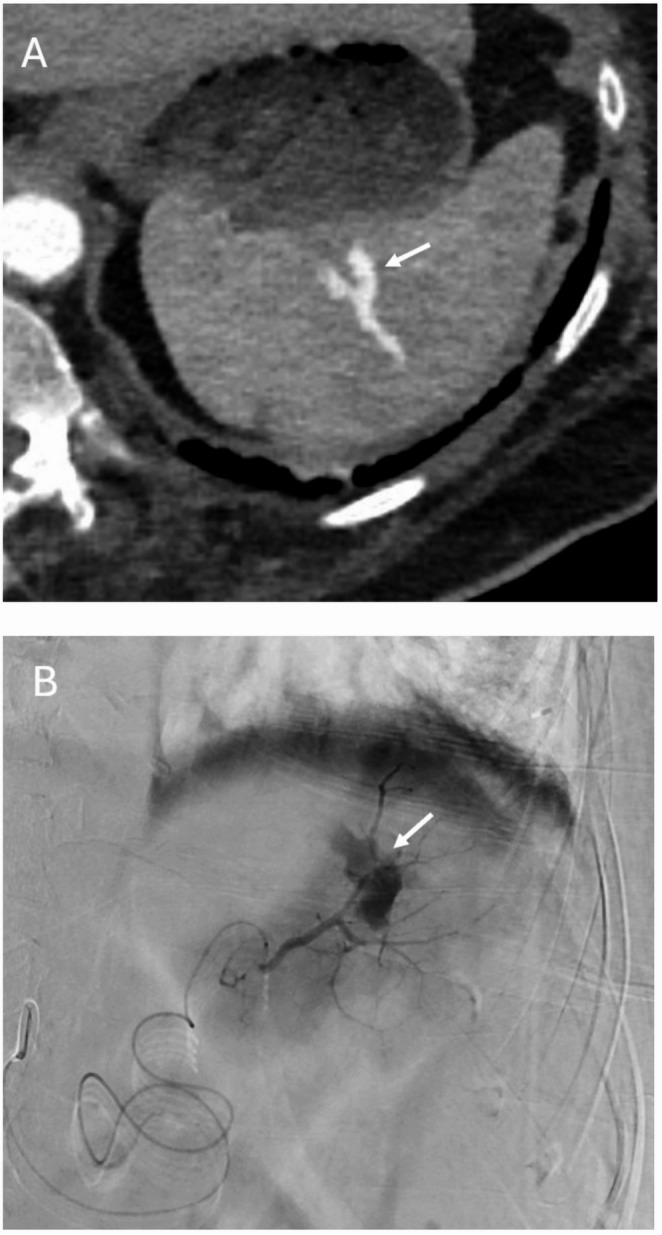
84-year-old female who fell down the stairs. Axial arterial phase CT (**A**) demonstrates an abnormal curvilinear contrast collection within the spleen (arrow), consistent with a pseudoaneurysm (AAST grade 4 injury) which was not mentioned in the original outside report. Subsequent angiography confirmed the presence of a traumatic splenic pseudoaneurysm (**B**, arrow) which was successfully treated with coil embolization (not shown)

**Fig. 3:**
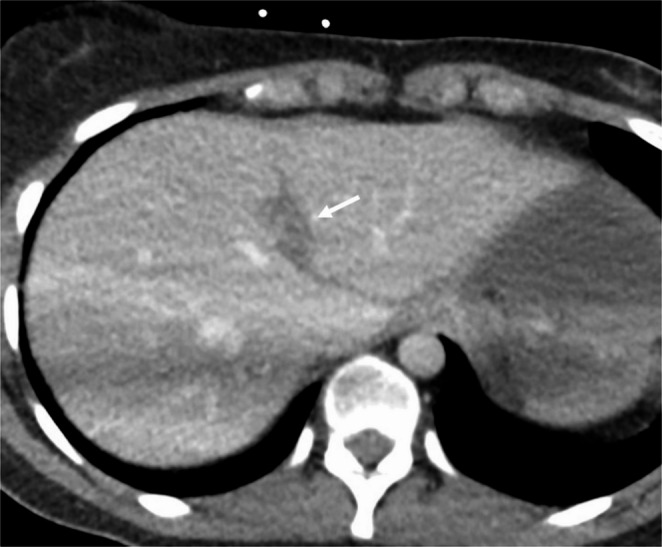
24-year-old female status-post motor vehicle collision. Axial post contrast CT demonstrates a laceration through the central liver (arrow) which was not described in the original outside report. The patient was managed conservatively

**Fig. 4:**
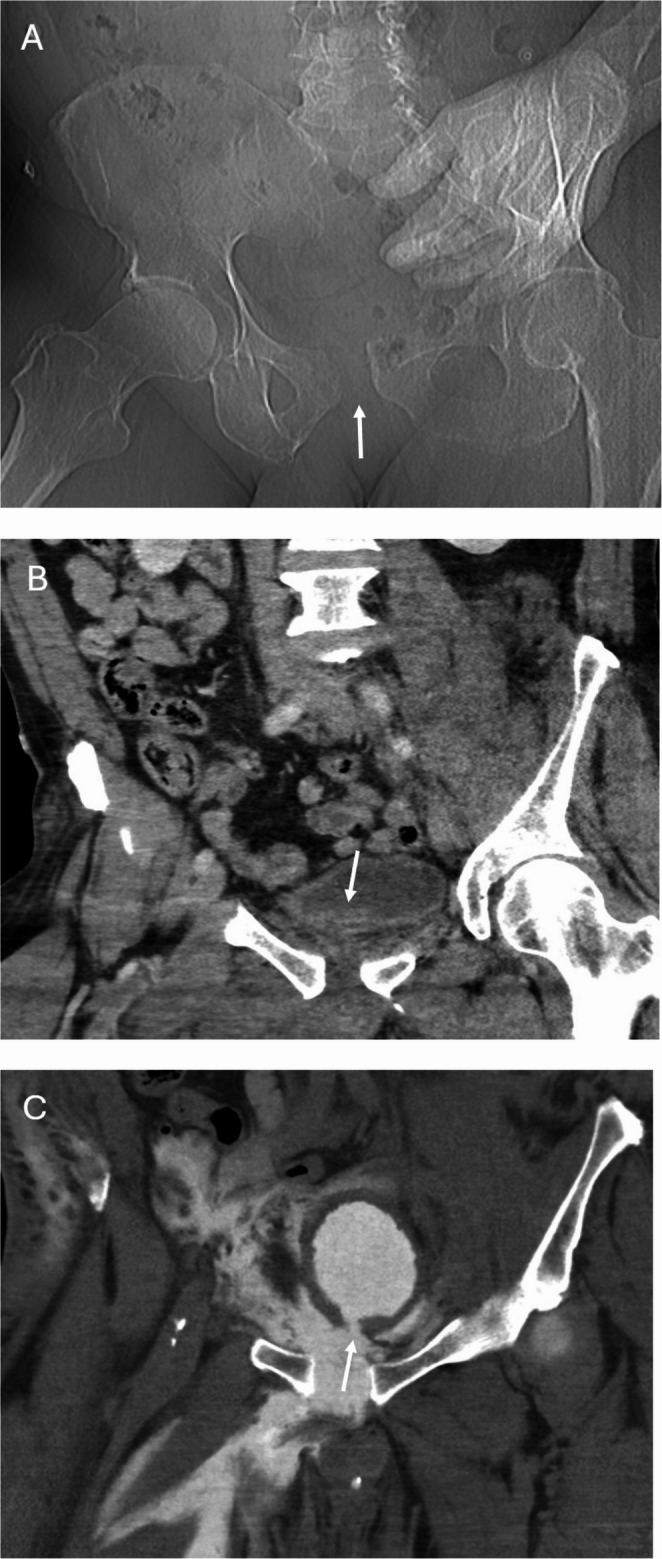
62-year-old male who was struck by a motor vehicle. CT scout image (**A**) demonstrates abnormal widening of the pubic symphysis. Coronal CT image (**B**) demonstrates a small hematoma with adjacent bladder wall irregularity (arrow), which was not mentioned in the original outside report. Subsequent coronal CT cystogram (**C**) images demonstrated a focal defect in the bladder wall (arrow) with leak of a large amount of bladder contrast consistent with an extra-peritoneal bladder injury which was treated non-operatively with bladder decompression

**Fig. 5:**
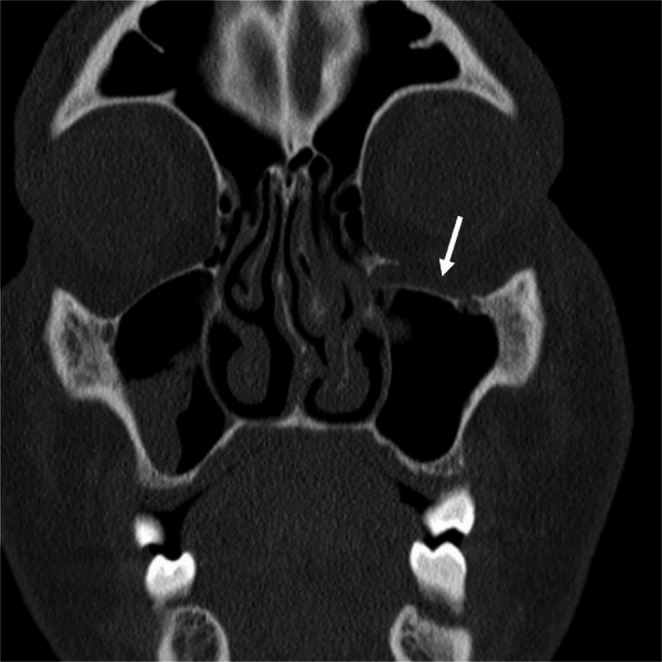
46-year-old male status post assault. Coronal CT of the face demonstrates a left orbital floor fracture (arrow) which was not described in the original outside report. The fracture was treated non-operatively

**Fig. 6:**
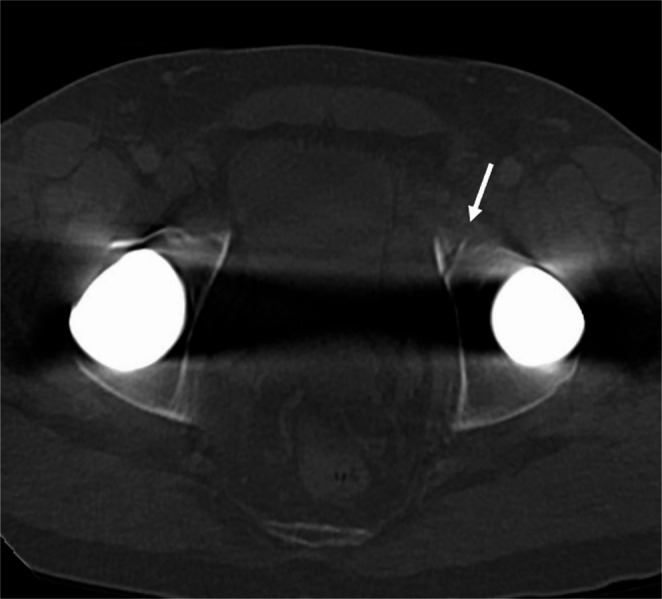
59-year-old male with a history of bilateral hip replacements presents with left hip pain status post ground level fall. There is a subtle left anterior acetabular fracture (arrow) that was not described in the original outside report. The patient was treated non-operatively

Of the 301 exams that were re-interpreted, 267 (88.7%) were CT exams, 31 (10.3%) were radiographic exams, and 3 (1.0%) were MRI exams. Cumulatively, a total of 75 exams (24.9%) had some type of discrepancy. There were major discrepancies in 26 exams (8.6%) that affected 25 patients (18.5%). There were an additional 49 minor discrepancies (16.3%) for 42 additional patients (31.1%, see Table 2 for details). The most common major discrepancies were mesenteric or bowel injuries (15.4%, Fig. 1), solid organ injury upgrades (11.5%, Figs. 2 and 3), fractures requiring fixation (11.5%), and urinary tract injuries (11.5%, Fig. 4). The most frequently encountered minor discrepancies were fractures not requiring further intervention (69.4%, Figs. 5 and 6) and soft tissue injuries (20.4%). Table 3 lists management changes for patients with major discrepancies. Of note, a total of 7 patients (5.2% of the total cohort) required either surgery or an interventional radiology procedure solely based on the results of the overread.

**Table 2:** Discrepancies

	Major discrepancy (%)	Minor discrepancy (%)	No change (%)
Exams (*N* = 301)	26 (8.6%)	49 (16.3%)	226 (75.1%)
Patients (*N* = 135)	25 (18.5%)	42 (31.1%)	68 (50.4%)

**Table 3:** Change in management for major discrepancies

Change in management	Total number of exams	Percent of major discrepancies (%)	Percent of all exams (%)
Surgical intervention	5	19.2	1.7
IR procedure	2	7.7	0.7
Further imaging studies	12	46.2	4.0
Admission for serial abdominal exams	3	11.5	1.0
Change in medical management	3	11.5	1.0
Joint reduction	1	3.8	0.3

*IR* interventional radiology

The mean ISS for the patients with major discrepancies (13.7) was higher than those with minor discrepancies (13.4), though the difference was not statistically significant (*p* = 0.8). The mean ISS for patients without a discrepancy was 12.9.

There was a statistically significant difference in the relationship between the presence of a discrepancy and mechanism of injury (*p* = 0.0427), driven by a disproportionate number of motor vehicle collisions (MVC, Table 4). There is a statistically significant association with a discrepancy following MVC compared to the other mechanisms of injury (*p* = 0.0228).

**Table 4:** Discrepancy vs. mechanism of injury

Mechanism of injury*#	No discrepancy (*N* = 226)	Minor discrepancy (*N* = 49)	Major discrepancy (*N* = 26)
MVC	70 (31.0%)	18 (36.7%)	15 (57.7%)
Fall	104 (46.0%)	19 (38.8%)	5 (19.2%)
Assault	20 (8.8%)	4 (8.2%)	1 (3.8%)
MCC	12 (5.3%)	3 (6.1%)	0
Other blunt mechanism	8 (3.5%)	2 (4.1%)	2 (7.7%)
ATV accident	6 (2.7%)	1 (2.0%)	1 (3.8%)
GSW	4 (1.8%)	0	0
Bicycle accident	1 (0.4%)	1 (2.0%)	1 (3.8%)
Crush Injury	1 (0.4%)	1 (2.0%)	0
Stab Wound	0	0	1 (3.8%)

*MVC* motor vehicle collision, *MCC* motorcycle collision, *NOS* not otherwise specified, *ATV* all terrain vehicle, *GSW* – gunshot wound

*Total number of discrepancies exceeds the total number of patients as some patients had more than one discrepancy.

#There is a statistically significant relationship between the presence of a discrepancy and mechanism of injury (*p* = 0.0427)

(%)Percent of patients with that particular discrepancy classification

## Discussion

Subspecialty radiology services at tertiary care referral centers are often requested to reinterpret exams performed on patients transferred from outside institutions. These reinterpretations, often called “overreads”, become part of the patient’s permanent medical record and are often requested to help direct patient management in the setting of oncologic, surgical, and trauma care [5, 10].

Previous studies have noted a discrepancy rate up to nearly 20% between the original report and the overread by emergency radiologists in the setting of trauma [5–10]. Other radiology subspecialties have made similar observations but with even higher discrepancy rates [3]. A systematic review by Wu et al., published in 2014, analyzed 46 studies focused on double reading of exams. They found that the pooled total discrepancy rate was 7.7% and the major discrepancy rate affecting patient management was 2.4%, though this varied by modality and body region [10]. A meta-analysis by Geijer & Geijer published in 2018 found a very wide discrepancy rate of 0.4 to 22%, though this varied by modality, body region, and subspecialty. They found that the pooled discrepancy rate was 7.7% and a major discrepancy rate of 2.4%. They also found that major discrepancy rates for head CT and spine CT (0.8% and 0.7%, respectively) were lower than those for chest CT and abdominal CT (2.8% and 2.7%, respectively). Interestingly, they found that the lack of blinding of the initial report was associated with a lower major discrepancy rate (2.0% vs. 12.1% for blind reading), suggesting that access to the original report can insert interpreter bias [6].

In our study, we found that nearly a quarter (24.9%) of imaging exams affecting nearly half (49.6%) of trauma patients had either a major or minor discrepancy in the original report, with a major discrepancy rate of 18.5%, which is at the higher end of the range that has been previously reported [3, 5–10]. The most commonly encountered discrepancies in our study were fractures or injuries to the bowel/mesentery or solid organs. While the most common change in management was obtaining further imaging studies, over one-quarter of major discrepancies (7 out of 25 [28%], or 2.3% of all imaging exam re-interpretations) resulted in either surgery or an interventional radiology procedure. Motor vehicle collisions, which accounted for 34% of all exams, accounted for 57% of major discrepancies (*p* = 0.0228 compared to non-MVC mechanism of injury). The greater discrepancy rate in MVC’s may reflect a greater overall complexity of the exams due to the presence of multiple simultaneous injuries and/or the presence of more subtle though clinically relevant findings (such as mesenteric or bowel injuries). Falls, which represented a greater percentage of all exams (42%), represented a lower percentage (19%) of all major discrepancies.

Our findings support the added value of emergency radiologist overreads for transferred trauma patients. This correlates with metanalyses that have shown that the overread is the more accurate interpretation approximately 90.5% of the time [3]. Our data adds to the growing body of literature supporting that discrepancies are greater in emergent patients than non-emergent patients, with rates of discrepancy reported as high as 19.7% in trauma populations in systematic reviews and as low as 2.4% in non-traumatic population systematic reviews [8, 9]. Despite increasing reports like ours, there is still a relative paucity of data regarding trauma patients transferred to level 1 trauma centers. This may be due to the institutional differences in overread procedures [5] or due to difficulties with reimbursement [6].

This study is subject to several limitations, as it is a one-year, retrospective review of imaging exam reinterpretation involving trauma patients transferred to a single level 1 trauma center. Access to the original outside report may introduce bias, as noted by Geijer & Geijer (Geijer 2014). Additionally, requesting a second opinion from a re-interpreting radiologist could also contribute to bias, since patients transferred to this institution are often more severely injured or require a higher level of care.

## Conclusions

We found that nearly a quarter (24.9%) of imaging exams affecting nearly half (49.6%) of trauma transfer patients had a discrepancy between the overread and the original report, resulting in a change in management in 18.5% of patients. Motor vehicle collisions were associated with more discrepancies compared to other mechanisms of injury. Of note, just over 5% of patients went on to surgery or an interventional radiology procedure because of the overread. The findings of our study continue to support the value of reinterpretation of outside imaging exams by dedicated emergency radiologists in the setting of trauma.

## Data Availability

Data cannot be shared openly to protect study participant privacy.

## References

[1] Lu MT, Tellis WM, Arvin DE (2014) Providing formal reports for outside imaging and the rate of repeat imaging. AJR Am J Roentgenol 203:107–111 24951202 10.2214/AJR.13.10617

[2] Robinson JD, McNeeley MF (2012) Transfer patient imaging: a survey of members of the American society of emergency radiology. Emerg Radiol 19:447–454 22527362 10.1007/s10140-012-1047-y

[3] Rosenkrantz AB, Duszak R, Babb JS, Glover M, Kang S (2018) Discrepancy rates and clinical impact of imaging secondary interpretations: a systematic review and meta-analysis. J Am Coll Radiol 15:1222–1231 30031614 10.1016/j.jacr.2018.05.037

[4] Vrablik M, Kessler R, Vrablik M, Mitchell S, Linnau K, Robinson J, Hippe D, Hall M (2021) Emergency radiology overreads change management of transferred patients with traumatic injuries. Ann Emerg Med 78(4):S35

[5] Flowers MG, Khatri GD, Robinson JD (2022) Transfer patient imaging: discordances between community and subspecialist emergency radiologists. Emerg Radiol 29(2):395–401 35041106 10.1007/s10140-022-02017-5

[6] Geijer H, Geijer M (2018) Added value of double reading in diagnostic radiology, a systematic review. Insights Imaging 9(3):287–301 29594850 10.1007/s13244-018-0599-0 PMC5990995

[7] Robinson JD, Kessler R, Vrablik ME et al (2022) Transfer patient imaging: assessment of the impact of discrepancies identified by emergency radiologists. J Am Coll Radiol 19(11):1244–1252 35973650 10.1016/j.jacr.2022.05.031 PMC10695447

[8] Robinson JD, Linnau KF, Hippe DS, Sheehan KL, Gross JA (2018) Accuracy of outside radiologists’ reports of computed tomography exams of emergently transferred patients. Emerg Radiol 25(2):169–173 29282579 10.1007/s10140-017-1573-8

[9] Robinson JD, Dahl A (2023) Transfer patient imaging: secondary interpretation reimbursement. Emerg Radiol 30(1):93–97 36477931 10.1007/s10140-022-02107-4

[10] Wu MZ, McInnes MDF, Macdonald DB, Kielar AZ, Duigenan S (2014) CT in adults: systematic review and meta-analysis of interpretation discrepancy rates. Radiology 270(3):717–735 24475832 10.1148/radiol.13131114

